# Supplementary material for: Structures and immune recognition of Env trimers from two Asia prevalent HIV-1 CRFs
Source: Nat Commun. 2023 Aug 4;14:4676. doi: 10.1038/s41467-023-40321-x (PMC10403546; doi:10.1038/s41467-023-40321-x)
Supplement: Supplementary file 1 — Supplementary Information [file 41467_2023_40321_MOESM1_ESM.pdf]

Structures and immune recognition of Env trimers from two Asia prevalent HIV-1 CRFs

Jun Niu[1,2,3,†], Qi Wang[1, †], Wenwen Zhao[1,†], Bing Meng[1,†], Youwei Xu[1,†], Xianfang Zhang[1], Yi Feng[4], Qilian Qi[1], Yanling Hao[4], Xuan Zhang[1], Ying Liu[4], Jiangchao Xiang[1], Yiming Shao[4,5,*], Bei Yang[1,6,*]

Correspondence to:
Bei Yang (yangbei@shanghaitech.edu.cn)
Yiming Shao (shaoyiming@cpl.ac.cn)

This PDF file includes:
Supplementary Figs. 1 to 9
Supplementary Tables 1 to 5
Supplementary References

Supplementary Figures

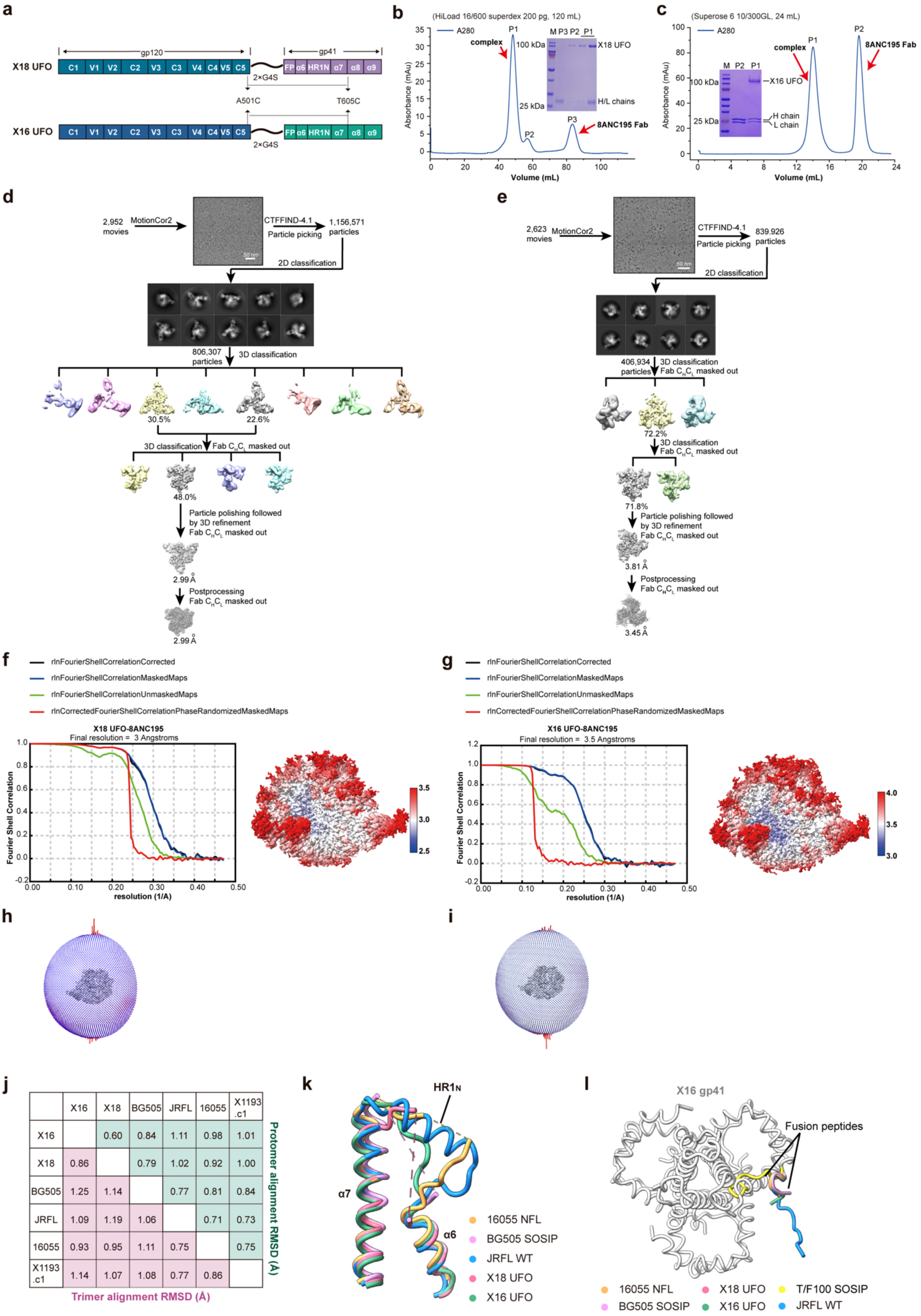

	X16	X18	BG505	JRFL	16055	X1193.c1
X16		0.60	0.84	1.11	0.98	1.01
X18	0.86		0.79	1.02	0.92	1.00
BG505	1.25	1.14		0.77	0.81	0.84
JRFL	1.09	1.19	1.06		0.71	0.73
16055	0.93	0.95	1.11	0.75		0.75
X1193.c1	1.14	1.07	1.08	0.77	0.86	

Supplementary Fig. 1 Cryo-EM structure determination of the X16 UFO-8ANC195 and X18

UFO-8ANC195 complexes. a Schematic diagram illustrating the X18 (CRF01_AE Env) and X16 (CRF07_BC Env) UFO design. **b-c** Gel filtration profiles of X18 UFO (b) or X16 UFO (c) in complex with 8ANC195 Fab. Shown in insets are the SDS-PAGE results of indicated peak fractions. The gel filtration and SDS-PAGE experiments were independently performed more than three times and representative results from one experiment are shown. **d-e** Schematics of cryo-EM data processing pipeline for X18 UFO-8ANC195 (**d**) and X16 UFO-8ANC195 (**e**). **f** The gold standard FSC curve showing the overall nominal resolution at 3.0 Å for X18 UFO-8ANC195 complex (left) and the cryo-EM map colored to local resolution (right). **g** The gold standard FSC curves showing the overall nominal resolution at 3.5 Å for X16 UFO-8ANC195 complex (left) and the cryo-EM map colored to local resolution (right). **h-i** Angular distribution (azimuth 0-120° are shown, C3 symmetry) of particles contributed to the final 3D reconstruction for X18 UFO-8ANC195 (**h**) and X16 UFO-8ANC195 (**i**). The length of cylinders represents the number of particles in the designated orientation. **j** Pair-wise root-mean-square deviations (RMSDs) among the Env trimers (lower left corner, pink) and Env protomers (upper right corner, green) representing different subtypes. **k** The conformations of $HR1_N$ in SOSIP (BG505 SOSIP, purple, PDB:5CJX)[1], NFL (16055 NFL, orange, PDB:5UM8)[2] and UFO (X18 UFO, salmon and X16 UFO, green) designs are compared to its native conformation (JRFL WT, blue, PDB:5FUU)[3]. **l** The FPs of X18 UFO (CRF01_AE, salmon) and X16 UFO (CRF07_BC, green) take on solvent exposed conformations as seen in BG505 SOSIP (clade A, purple, PDB:5CJX) or JRFL native (clade B, blue, PDB: 5FUU) or 16055 NFL (clade C, orange, PDB:5UM8) Env trimers, rather than buried conformation as seen in T/F100 SOSIP Env trimer (CRF01_AE, yellow, PDB:6NQD)[4]. Only gp41 of X16 UFO is shown as light grey ribbons for clarity. Source data are provided as a Source Data file.

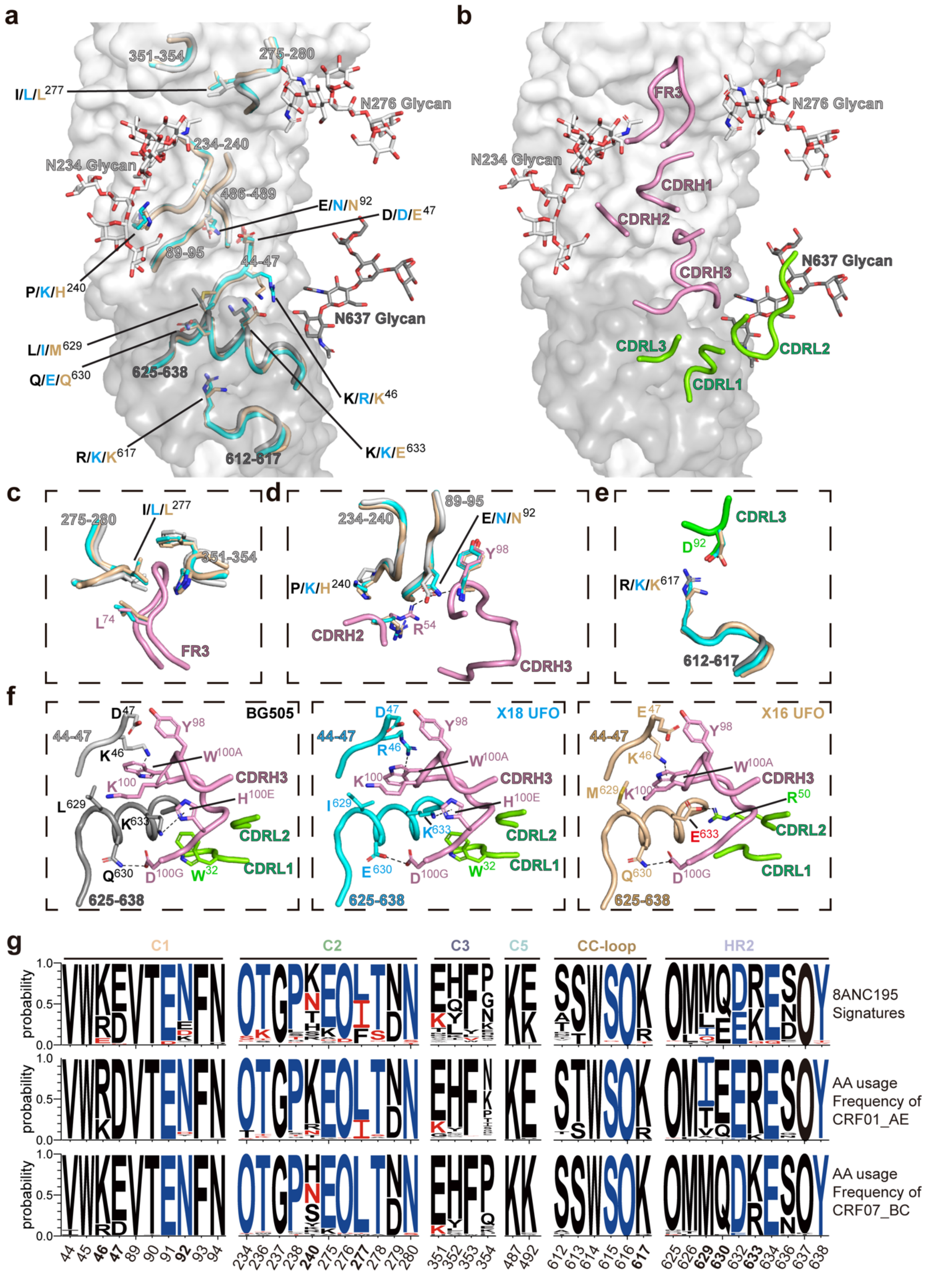

Supplementary Fig. 2 Comparison of the interactions between 8ANC195 and BG505 Env, X18 UFO or X16 UFO. a 8ANC195-contacting N234, N276 and N637 glycans are shown as stick models, other 8ANC195-contacting Env regions are shown as grey (BG505), cyan (X18

UFO) or wheat (X16 UFO) ribbons on surface presentations of HIV-1 Env protomer (gp120: light grey, gp41: dark grey) and labeled. 8ANC195-contacting residues that differ among BG505 Env, X18 UFO and X16 UFO are shown as stick models and labeled. **b** The complementarity-determining regions (CDRs) and framework region 3 (FR3) of 8ANC195 are shown as ribbons to illustrate their relative positions on Env surface. **c-f** Close-up views of the interfaces between 8ANC195 and indicated regions on BG505 Env (grey), X18 UFO (cyan) and X16 UFO (wheat). Env residues that differ among BG505 Env, X18 UFO and X16 UFO and their interacting residues in 8ANC195 are shown as stick models, labeled, and colored as in **a** and **b**. Cation-pi, polar and electrostatic interactions are indicated with black dashed lines. **c-e** Replacement of I277 (c), E92 and P240 (d), and R617 (e) in BG505 Env by L277, N92 and K240/H240, and K617 in X18 UFO and X16 UFO does not compromise the interactions between indicated regions. **f** Replacement of K46, D47, L629 and Q630 in BG505 Env (left) by R46, E47, I629/M629 and E630 in X18 UFO (middle) and X16 UFO (right) does not compromise their interactions with $W100^{A}$, Y98, K100 and $D100^{G}$ from 8ANC195 respectively. Replacement of K633 in BG505 Env (left) by E633 (labeled in red to highlight) in X16 UFO (right) breaks its original packing with $H100^{E}$ (CDRH3) and W32 (CDRL1) but enables new interaction with R50 (CDRL2) from 8ANC195. **g** WebLogo plots of the AA frequencies in 8ANC195-contacting regions indicate that the sensitivities of CRF01_AE (middle, n=618) and CRF07_BC (bottom, n=56) viruses to bNAbs 8ANC195 would not significantly differ from other M-group viruses (top). Env sequences and AAs signatures associated with sensitivity (blue) and resistance (red) to 8ANC195 (calculated by Fisher's test from n=262 neutralization dataset) are from the Los Alamos HIV sequence database (www.hiv.lanl.gov).

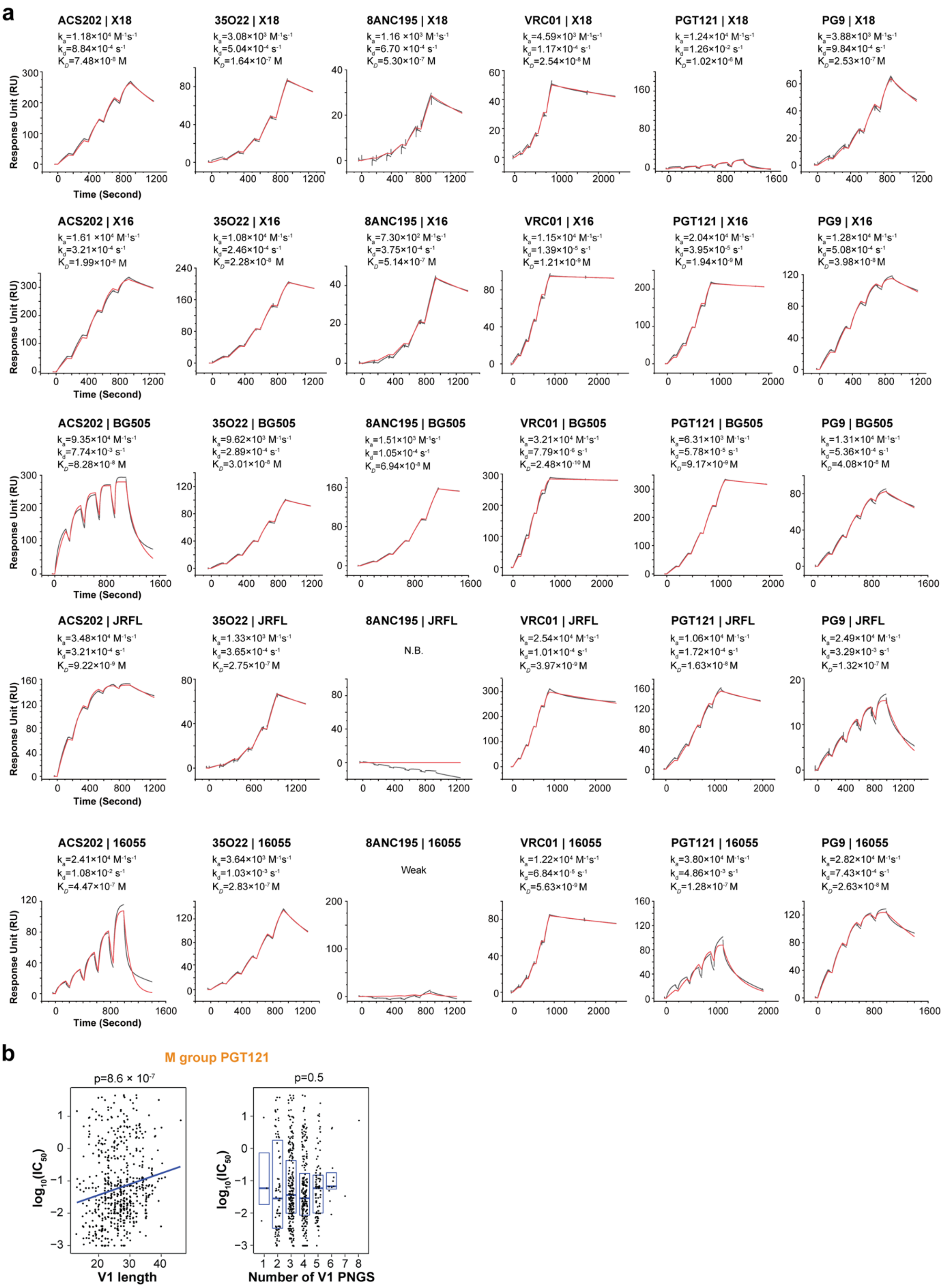

Supplementary Fig. 3 SPR binding curves of indicated bNAbs to immobilized X18, X16, BG505, JRFL and 16055 Env trimers. **a** All Envs used are in SOSIP design as UFO design would interfere with FP bNAbs binding. SPR binding curves are shown as black lines and the best

fits of the data to a 1:1 binding model are shown in red. All SPR experiments were independently performed at least three times and representative profiles from one experiment are shown. **b** Correlations between V1 loop characteristics and bNAb PGT121 sensitivities are represented by scatter plots. Top: Regression lines were fitted using the ggplot2 package in R (stat_smooth function). Bottom: The box represents the 25%-75% percentile and the median is shown as bold line. HIV-1 viruses neutralization data and V1 loop characteristics of corresponding viruses for PGT121 are prepared as described in Methods and stored in Source Data. The *p* values are calculated from Kendall's tau. The sample size is n=611 biologically independent samples. Source data are provided as a Source Data file.

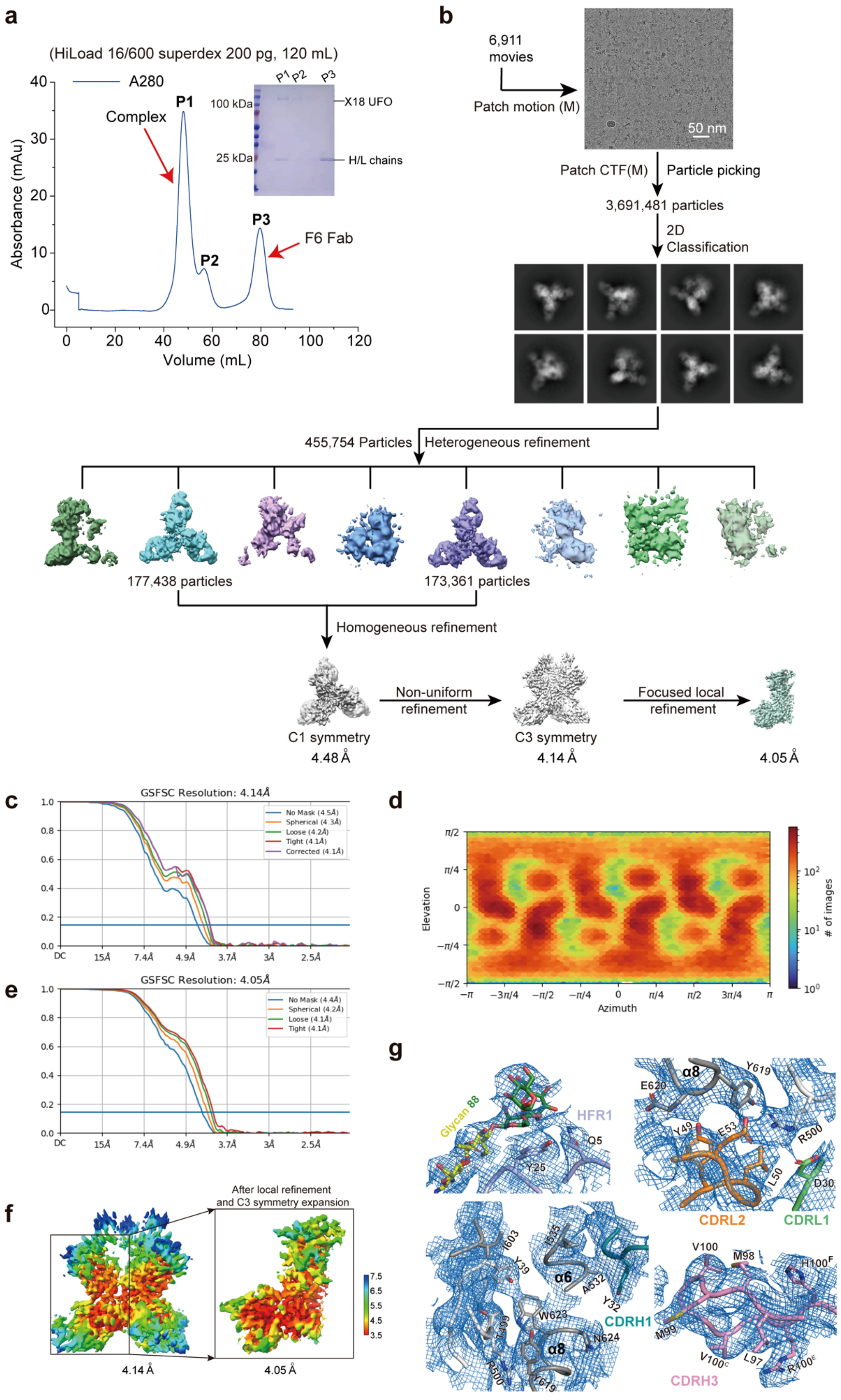

Supplementary Fig. 4 Cryo-EM structure determination of the X18 UFO-F6 complex. a Gel filtration profile of X18 UFO in complex with F6 Fab. Shown in insets is the SDS-PAGE result of

indicated peak fractions. The gel filtration and SDS-PAGE experiments were independently performed more than three times and representative results from one experiment are shown. **b** Schematic of cryo-EM data processing pipeline. **c** The gold standard FSC curve showing the overall nominal resolution at 4.14 Å for X18 UFO-F6 (trimer) complex after non-uniform refinement. **d** The angular distributions of all F6-X18 UFO particles used in the non-uniform refinement are shown as a heatmap. **e** The gold standard FSC curve showing the nominal resolution at 4.05 Å for X18 UFO-F6 (protomer) complex after interface-focusing local refinement (the V1/V2/V3 regions of X18 and the constant regions of F6 Fab are both omitted by applying a soft mask) and symmetry expansion. **f** Final 3D density map colored according to the local resolutions. **g** Densities at the interface between X18 UFO and F6 after local refinement. Source data are provided as a Source Data file.

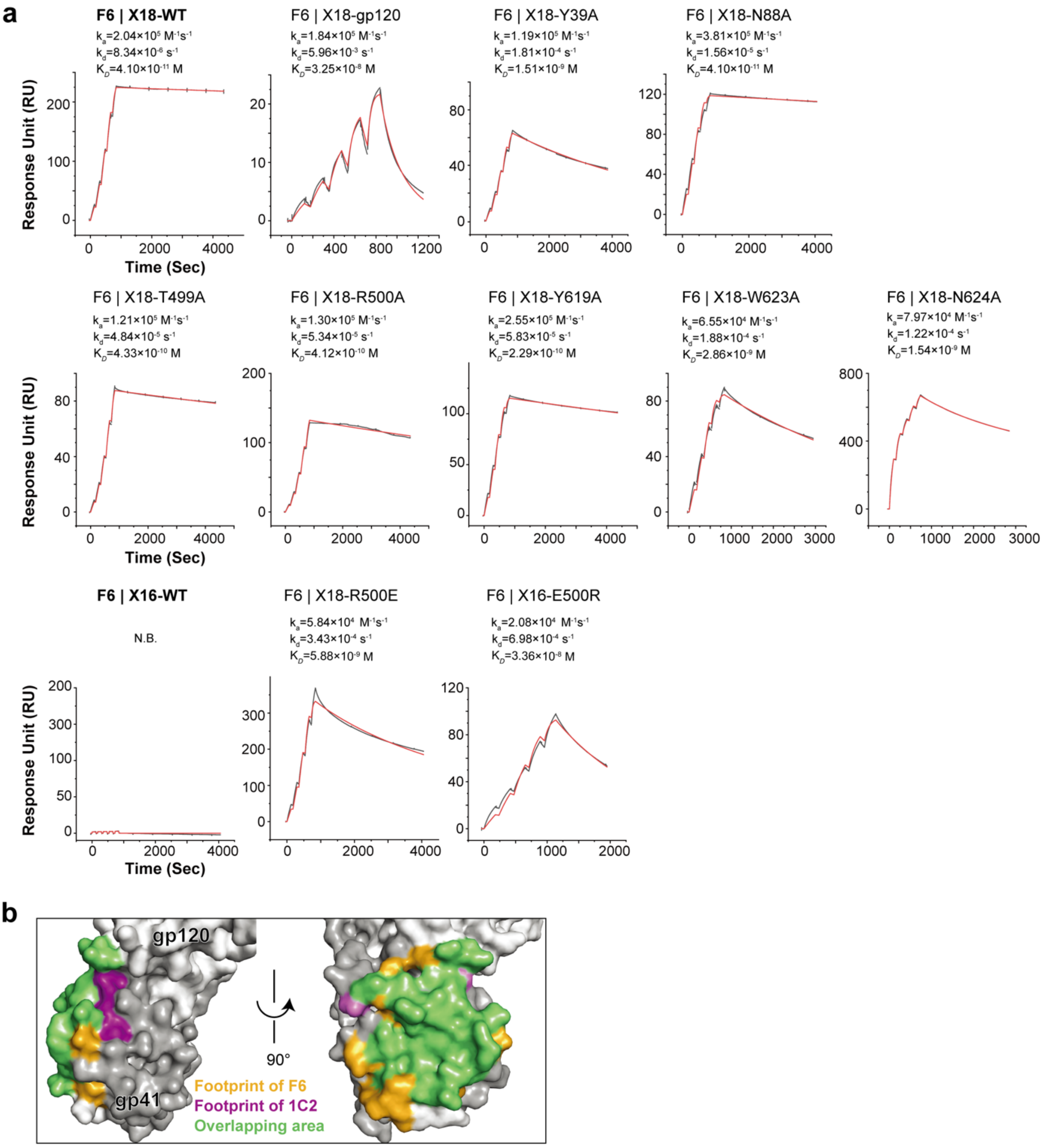

Supplementary Fig. 5 SPR binding curves of F6 to immobilized X18 UFO, X16 UFO, and their mutants. a SPR binding curves are shown as black lines and the best fits of the data to a 1:1 binding model are shown in red. All SPR experiments were independently performed at least three times and representative profiles from one experiment are shown. **b** Footprint of F6 on HIV-1 Env (orange) partially overlaps the potential epitope of 1C2 (purple), overlapping area is colored green.

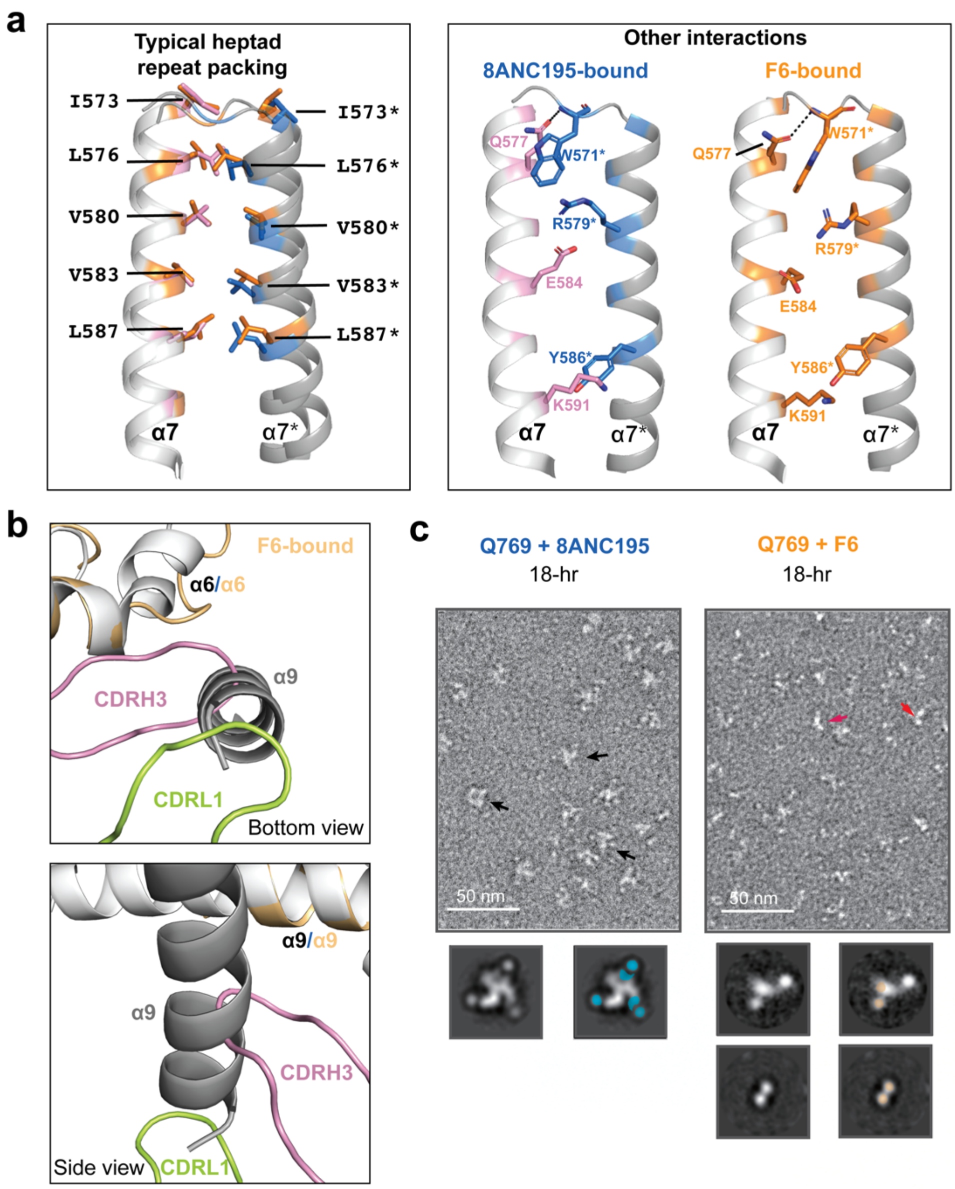

Supplementary Fig. 6 F6 binding induces trimer disassembly. a Compared to the 8ANC195-bound state, the interactions among α7 helices (Left: typical heptad repeat packing; Right: other interactions) remain largely unchanged in the F6-bound state. **b** Binding of F6 would clash with the C-terminus of the α9 helix. **c** F6 binding also induces disassembly of Q769 (clade A) Env trimers. nsEM micrographs of 8ANC195-bound (left) and F6-bound (right) Q769 UFO at 18-hr. Representative Fab-bound Env trimers and Fab-bound Env protomers are pointed with black and red arrows respectively. Shown below each nsEM micrograph are the representative 2D class averages of corresponding samples and the locations of Fabs in each class are highlighted with blue (8ANC195) or orange (F6) blocks on the right.

a

Binders	Envs	k_a ($M^{-1}s^{-1}$)	k_d (s^{-1})	K_D (M)	Relative affinity
**PGT145**	X18 UFO	2.23×10^{4}	8.17×10^{-4}	3.67×10^{-8}	100%
	X18 UFO + F6 (RT, 1-hr)	1.06×10^{3}	3.21×10^{-4}	3.02×10^{-7}	12.2%
**PG9**	X18 UFO	1.66×10^{3}	4.45×10^{-4}	2.67×10^{-7}	100%
	X18 UFO + F6 (RT, 1-hr)	3.64×10^{2}	6.36×10^{-4}	1.75×10^{-6}	15.3%

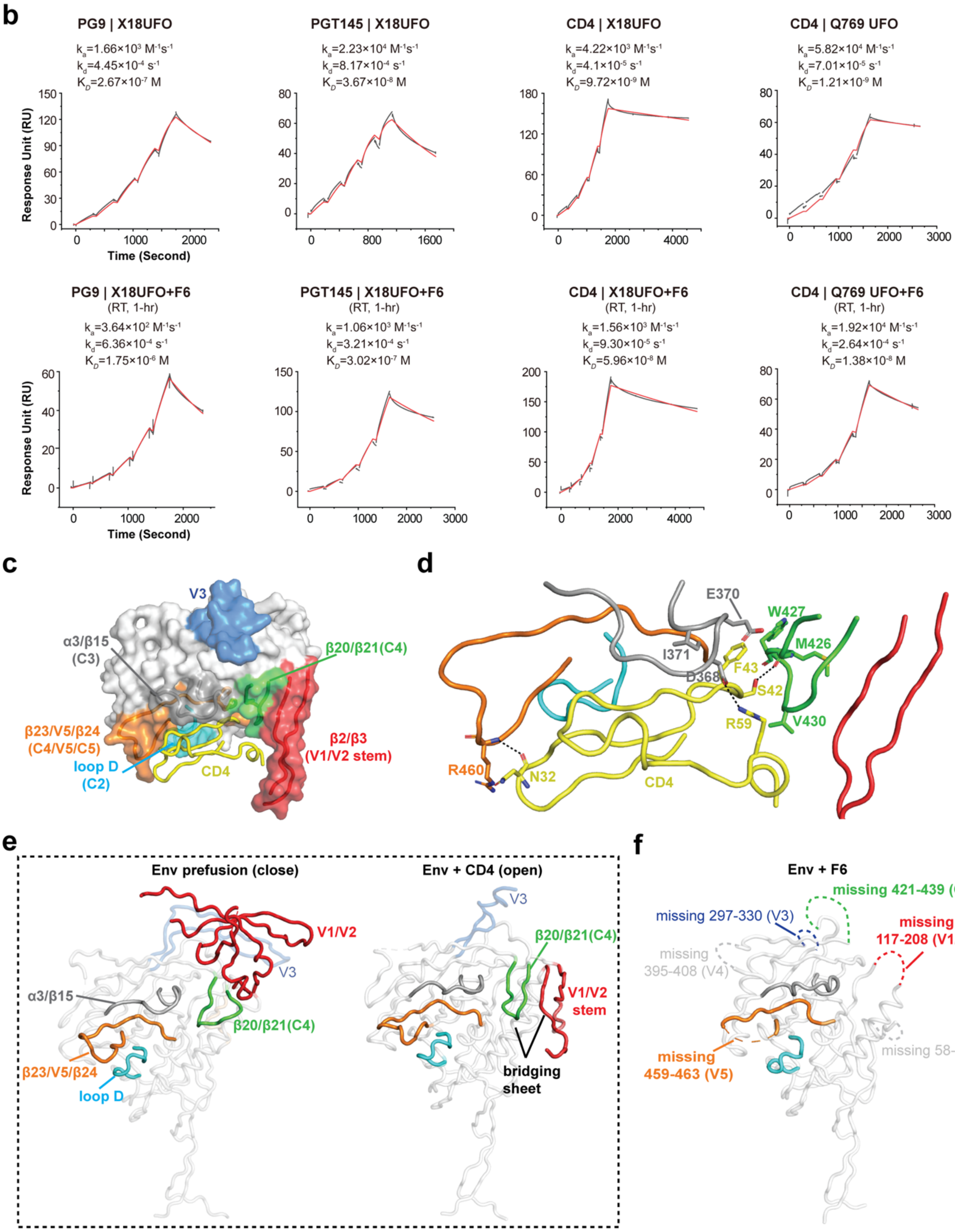

Supplementary Fig. 7 F6-induced destabilization of Env apex proximal regions hinders the

binding of apex bNAbs and CD4 receptor. a-b Interaction kinetics (**a**) and SPR sensorgrams (**b**) of indicated Envs or F6-bound Envs to PG9, PGT145 or sCD4. F6-bound Envs are formed by incubating X18 UFO (or Q769 UFO) with F6 for 1 hr. SPR sensorgrams are shown as black lines and the best fits of the data are shown in red. All SPR experiments were independently performed at least twice and representative profiles from one experiment are shown. **c** CD4 (yellow ribbons) is embraced by a depression formed by five gp120 segments on the surface of Env trimer: β2/β3 (V1/V2 stem, red), loop D (C2, cyan), α3/β15 excursion (C3, grey), β20/β21 hairpin (C4, green) and β23/V5/β24 (C4/V5/C5, orange)[5, 6]. **d** Interactions between CD4 and β20/β21 hairpin or 459-463aa of V5 are shown in detail, while other interactions between CD4 and gp120 are not shown for clarity. Main chain interactions are observed between $N32^{CD4}$ and $R460^{V5}$. $F43^{CD4}$ and $R59^{CD4}$ are two CD4 residues critical for Env binding, and they form hydrophobic interactions with W427 and V430 from the β20/β21 hairpin respectively. Meanwhile, $S42^{CD4}$ forms H-bond with the main chain oxygen of M426 from the β20/β21 hairpin. **e** Side-by-side comparison of gp120 in prefusion close state (left) and CD4-bound open state (right). In the presence of CD4, the V1/V2 regions (red) and β20/β21 (green) of Env rearrange themselves to form the bridging sheet[6], which in turn keeps key Env residues like $M426^{\beta20/\beta21}$, $W427^{\beta20/\beta21}$ and $V430^{\beta20/\beta21}$ at proper positions to interact with CD4 (**d**). **f** F6 binding induces destabilization of Env apex proximal regions, including spans of 117-208, 297-330, 421-439 and 459-463 from V1/V2 (red dashes), V3 (blue dashes), C4 (green dashes) and V5 (orange dashes) respectively. Given the key roles of V1/V2 stem, β20/β21 hairpin (C4) and V5 loop in CD4 engagement (**c**, **d**), F6-induced destabilization of apex proximal regions would impede the binding of CD4. **c-e** The structure of CD4-bound Env are based on PDB: 5VN3.

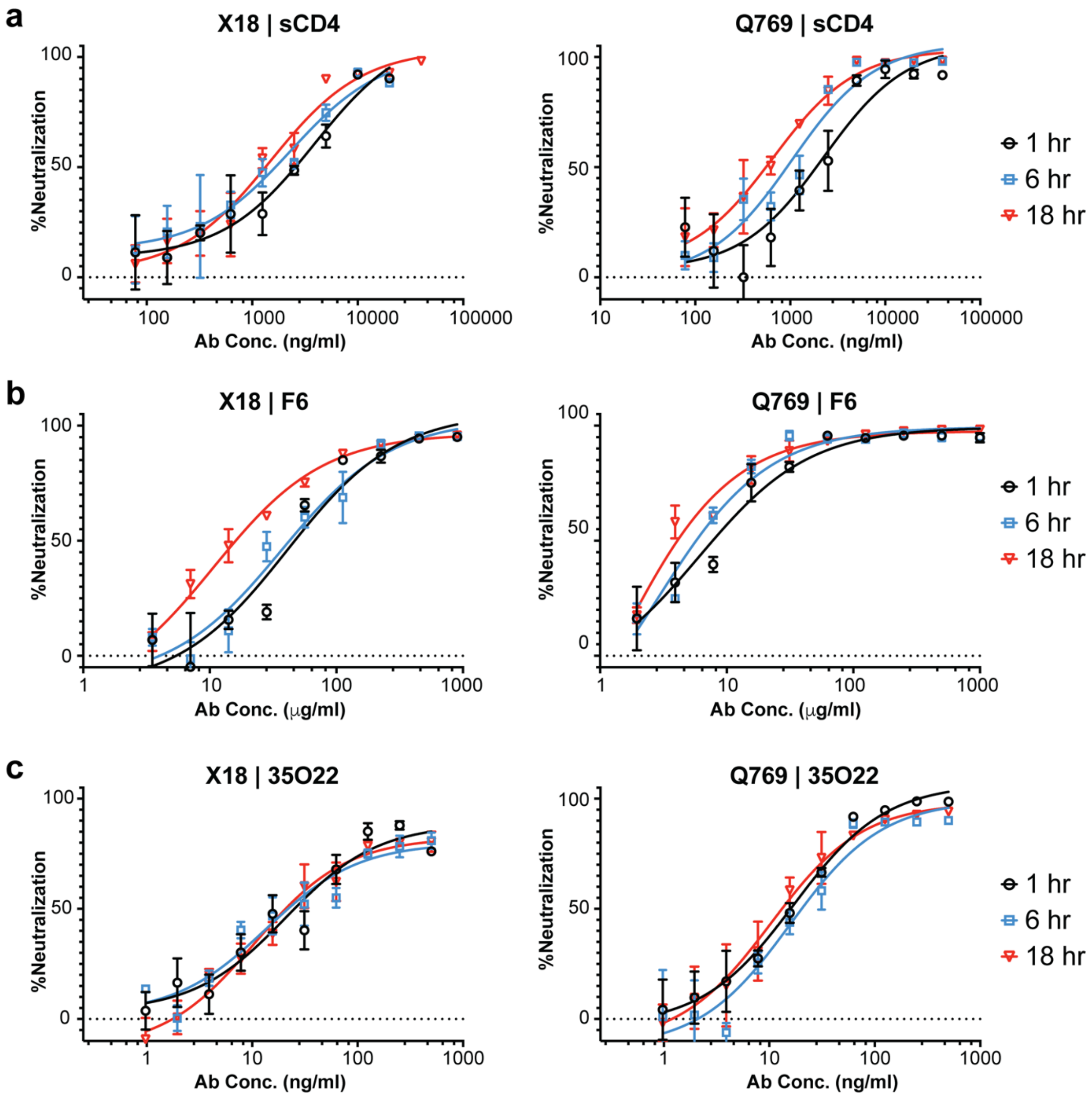

Supplementary Fig. 8 Neutralization profiles for sCD4, F6 IgG and 35O22 IgG in pre-incubation neutralization assay. a-c X18 (left) or Q769 (right) PsV was incubated with sCD4 (**a**), F6 IgG (**b**) and 35O22 IgG (**c**) for various periods, before being added to TZM-bl cells to test for infectivity. Neutralizing activities are represented as mean ± SD (n=3 biologically independent experiments).

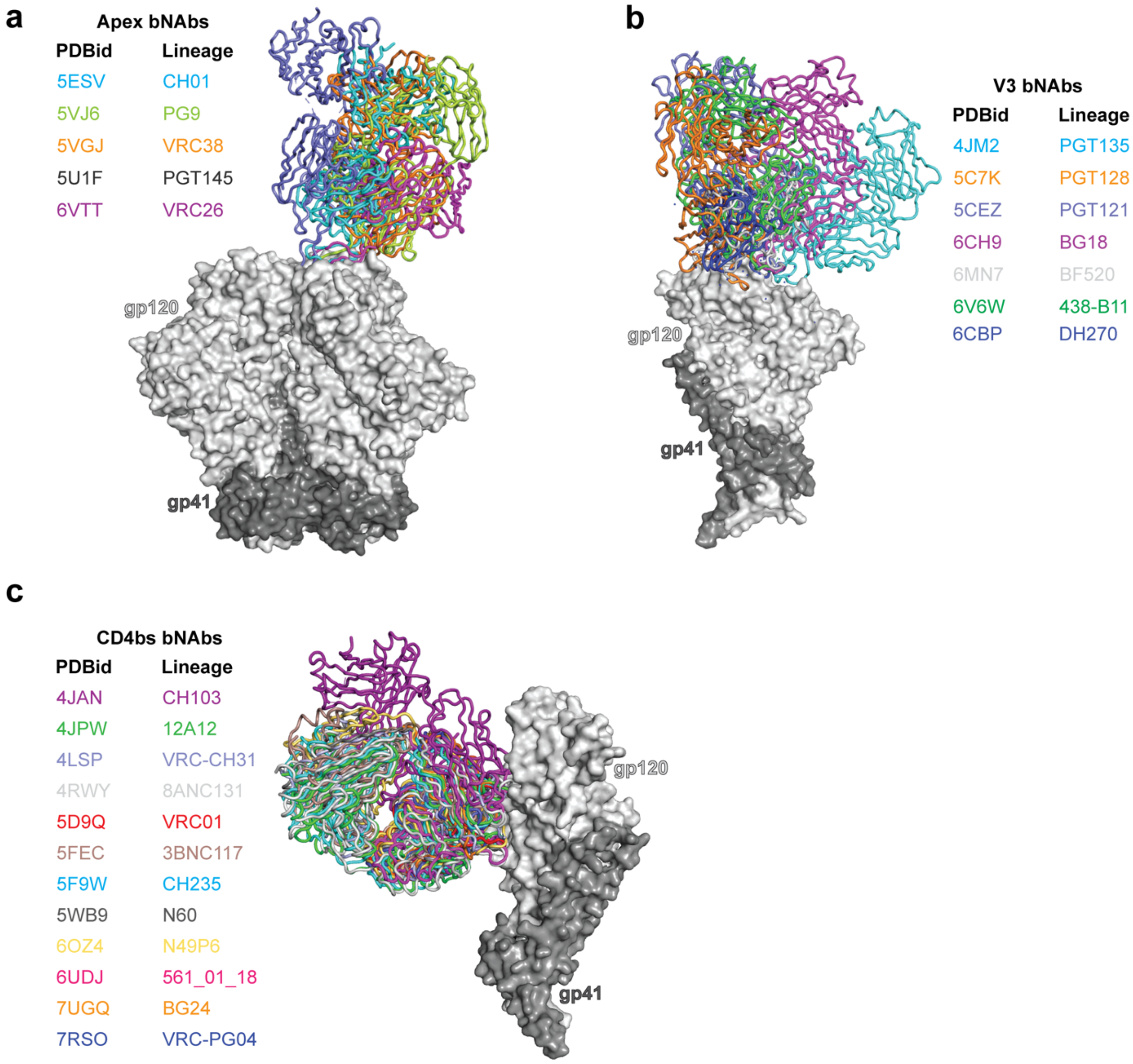

Supplementary Fig. 9 The convergence of V2 apex, V3 glycan or CD4bs bNAbs' epitopes. a-c bNAbs targeting the V1/V2 apex[7-11] (**a**), V3[12-18] (**b**) or CD4bs[19-30] (**c**) frequently target overlapping epitopes, although they belong to different clonal lineages and are from different donors. The representative bNAbs of each clonal lineage are shown as ribbons and colored as indicated, while Env trimer (**a**) or protomer (**b, c**) is shown as surface presentation. The PDBid of each bNAb is also shown.

Supplementary Table 1. Cryo-EM data collection, model refinement and validation statistics.

	X16 UFO-8ANC195 (EMDB-34194) (PDB 8GPJ)	X18 UFO-8ANC195 (EMDB-34193) (PDB 8GPI)	X18 UFO-F6 (EMDB-34192) (PDB 8GPG)	X18 UFO protomer-F6 VHVL domain (EMDB-34190) (PDB 8GP5)
**Data collection and processing**				
Magnification	22,500	22,500	22,500	22,500
Voltage (kV)	300	300	300	300
Electron exposure (e^{-}/Å^{2})	50	50	50	50
Defocus range (μm)	-0.8 to -2.5	-0.8 to -2.5	-0.8 to -2.5	-0.8 to -2.5
Pixel size (Å)	1.06	1.06	1.06	1.06
Symmetry imposed	C3	C3	C3	C1
Initial particle projections (no.)	839,926	1,156,571	3,691,481	3,691,481
Final particle projections (no.)	210,695	205,630	350,799	1,052,397
Map resolution (Å)	3.5	3.0	4.1	4.1
FSC threshold	0.143	0.143	0.143	0.143
Map resolution range (Å)	2.8-5.0	2.8-5.0	3.5-6.0	3.5-6.0
**Refinement**				
Initial model used (PDB code)	5CJX	5CJX	8GPI	8GPG
Model resolution (Å)	3.5	3.1	4.3	4.3
FSC threshold	0.5	0.5	0.5	0.5
Map sharpening B factor (Å^{2})	DeepEMhancer	DeepEMhancer	-157.0	-200.1
Model composition				
Non-hydrogen atoms	20,259	20,265	15,864	5,470
Protein residues	2,382	2,364	1,920	646
Ligands	BMA: 13, NAG: 82, MAN: 31	BMA: 9, NAG: 93, MAN: 30	BMA: 3, NAG: 39, MAN: 6	BMA: 2, NAG: 20, MAN: 4
R.m.s. deviations				
Bond lengths (Å)	0.005	0.004	0.004	0.004
Bond angles (°)	0.666	0.566	0.686	0.672
B factors (Å^{2}, mean value)				
Protein residues	79.1	62.9	88.9	87.4
Ligands	87.4	72.4	104.0	112.4
**Validation**				
MolProbity score	1.84	1.32	2.05	1.97
Clashscore	9.98	5.14	13.04	12.13
Rotamer outliers (%)	0	0	0	0
Ramachandran plot				
Favored (%)	95.3	97.8	93.5	94.5
Allowed (%)	4.7	2.2	6.5	5.5
Disallowed (%)	0	0	0	0

Supplementary Table 2. X-ray data collection and refinement statistics of F6 Fab.

	**F6 Fab (PDB:8GPK)**
**Data collection statistics**	
Beamline	BL19U1
Wavelength (Å)	0.9787
Space group	$P12_11$
Cell dimensions	
a, b, c (Å)	91.2, 62.3, 97.8
α, β, γ (°)	90.0,105.2, 90.0
Resolution range (Å)*	19.7 - 3.3 (3.5 - 3.3)
No. of observed reflections	103,727 (4,828)
No. of unique reflections	15,277 (1,352)
Completeness (%)*	97.7 (87.2)
R_{meas} (%)[*,a]	24.0 (52.0)
R_{pim} (%)[*, b]	13.0 (29.0)
$\langle I/\sigma(I)\rangle$*	6.1 (3.4)
$CC_{1/2}$[*,c]	0.97 (0.93)
Redundancy*	6.6 (6.4)
Wilson B (Å^2)	63.6
**Refinement statistics**	
Reflections used in refinement*	15,269 (1,351)
Reflections used for R_{free}*	1,533 (144)
R_{work} (%)[*,d]	24.2 (31.8)
R_{free} (%)[*,d]	28.8 (34.7)
Atoms	
Protein	5,765
Average B-values (Å^2)	
Protein	73.28
RMSD bond length (Å)	0.004
RMSD bond angle (°)	0.73
Ramachandran favored (%)	96.3
Ramachandran allowed (%)	3.7

*Statistics for the highest-resolution shell are shown in parentheses.

[a] $R_{meas} = \frac{\sum_{hkl}\left[\frac{N(hkl)}{N(hkl)-1}\right]^{\frac{1}{2}} \times \sum_i |I_i(hkl) - \langle I(hkl)\rangle|}{\sum_{hkl}\sum_i I_i(hkl)}$

[b] $R_{pim} = \frac{\sum_{hkl}\left[\frac{1}{N(hkl)-1}\right]^{\frac{1}{2}} \times \sum_i |I_i(hkl) - \langle I(hkl)\rangle|}{\sum_{hkl}\sum_i I_i(hkl)}$

[c] $CC_{\frac{1}{2}} = \frac{\sum(x-\langle x\rangle)(y-\langle y\rangle)}{[\sum(x-\langle x\rangle)^2 \sum(y-\langle y\rangle)^2]^{\frac{1}{2}}}$

[d] $R_{work} = \frac{\sum||F_{obs}|-|F_{calc}||}{\sum|F_{obs}|}$; R_{free} is defined as R_{work} calculated from 10% of the reflections that were excluded from refinement.

Supplementary Table 3. Statistics of intact and disassembled Env trimer particles at different time points.

	X18-F6			X18-8ANC195		
	**1-hr**	**7-hr**	**18-hr**	**1-hr**	**7-hr**	**18-hr**
**Non-trimer particle numbers**	1733	5112	9450	515	269	1027
**Trimer particle numbers**	8145	4787	785	3214	2344	5272
**Total particle numbers**	9878	9899	10235	3729	2613	6299
**Env trimer percentage (%)**	82.46%	48.36%	7.67%	86.19%	89.71%	83.70%

Supplementary Table 4. gp120 shedding from X18 and Q769P PsVs.

		% gp120 associated with virions*					
		X18			Q769		
		repeat-1	repeat-2	repeat-3	repeat-1	repeat-2	repeat-3
CD4	0 hr	90.50%	95.57%	89.52%	94.55%	92.35%	92.32%
	1 hrs	80.91%	82.99%	80.13%	69.41%	69.70%	64.52%
	6 hrs	68.16%	74.73%	68.25%	39.01%	33.14%	36.50%
	18 hrs	49.58%	61.23%	53.62%	27.43%	26.28%	25.56%
F6	0 hr	98.84%	101.57%	97.57%	101.73%	102.73%	95.95%
	1 hrs	98.33%	93.97%	96.95%	98.57%	100.21%	103.56%
	6 hrs	96.47%	98.02%	95.61%	100.05%	101.88%	102.78%
	18 hrs	99.21%	97.94%	95.25%	95.28%	96.41%	91.96%
35O22	0 hr	99.13%	101.40%	98.07%	100.57%	97.30%	93.20%
	1 hrs	99.31%	98.91%	98.90%	96.57%	99.93%	100.04%
	6 hrs	101.21%	100.98%	97.38%	95.87%	108.87%	102.69%
	18 hrs	100.47%	100.46%	99.78%	97.67%	99.46%	93.98%

* Percentage of gp120 associtated with virions was calculated with the equation:

$$\frac{gp120\ associated\ with\ virions}{gp120\ in\ supernatant + gp120\ associated\ wth\ virions}$$

and normalized to samples incubated for the same periods without ligand

Supplementary Table 5. Oligos used for plasmids construction.

769_deltaCT_R1	AGCATGGTATCATTAGTTGATCACGCTCAGCACGG
769_deltaCT_F1	CTGTGGCGATGGGGTACTATGATTTTGGGGATGATCCTGATCTGCTGCTC
X18_deltaCT-R1	GAGCATGGTATCATTAATTCACGATGCTCAGCACAGCAAAAATG
X18_deltaCT-F1	TTGGCAAAGCACATGAGAGTGAGGGGGATACAGATGAATTG
X18_Y39A_R	ACACCGTAGGCGACTGTAACCCACAAATTGTTAGATCTAGAGC
X18_Y39A_F	TTACAGTCGCCTACGGTGTTCCTGTCTGGCG
X18_N88A_F	CCACAAGAAATACCTCTCGAGGCTGTTACCGAG
X18_N88A_R	TGTTCTTCCACATGTTGAAATTCTCGGTAACAGCCTCGAGAGGTATTTCT
X18_T499A_R	TCGACTACCCGCCGCTTACATCTGGCAGGCGCAATGCCAAGAGGC
X18_T499A_F	TGTAAGCGGCGGGTAGTCGATGG
X18_R500A_R	TCGACTACCCGCCGCTTACAGGCGGTAGGCGCAATGCCAAGAGGC
X18_R500A_F	CCGCCTGTAAGCGGCGGGTAGTCGATGG
X18_Y619A_R	TATCTCTTCAGCGCTTTTATTTGACCACGTACTGTTC
X18_Y619A_F	ATAAAAGCGCTGAAGAGATATGGAATAACATGACCTG
X18_W623A_F	AACATGACCTGGATAGAGTGGGAAAAGGAAATAAG
X18_W623A_R	CACTCTATCCAGGTCATGTTATTGGCTATCTCTTCATAGCTTTTATTTGAC
X18_N624A_R	TCGACTACCCGCCGCTTACATCTGGTAGGCGCAATGCCAAGAGGC
X18_N624A_F	TTGCGCCTACCAGATGTAAGCGGCGG
X18_gp120_R	CTAGCATGATGATGATGATGATGATCGACTACCCGCCGCTTAC
X18_gp120_F	TCACCGTCGTCGACGCCACCATGGACGCAATGAAACGAGGAC
X18_R500E_R	GCTTACATTCGGTAGGCGCAATGCCAAGAGG
X18_R500E_F	CGCCTACCGAATGTAAGCGGCGGGTAGTCG
X16_E500R_R	GTTTACACCGGGTCGGGGCCACACCG
X16_E500R_F	CCCGACCCGGTGTAAACGCAGAGTTGTTGAAGGGG

Supplementary References

1. Scharf L, Wang H, Gao H, Chen S, McDowall AW, Bjorkman PJ. Broadly Neutralizing Antibody 8ANC195 Recognizes Closed and Open States of HIV-1 Env. *Cell* **162**, 1379-1390 (2015).

2. Guenaga J*, et al.* Glycine Substitution at Helix-to-Coil Transitions Facilitates the Structural Determination of a Stabilized Subtype C HIV Envelope Glycoprotein. *Immunity* **46**, 792-803 e793 (2017).

3. Lee JH, Ozorowski G, Ward AB. Cryo-EM structure of a native, fully glycosylated, cleaved HIV-1 envelope trimer. *Science* **351**, 1043-1048 (2016).

4. Ananthaswamy N*, et al.* A sequestered fusion peptide in the structure of an HIV-1 transmitted founder envelope trimer. *Nat Commun* **10**, 873 (2019).

5. Kwong PD, Wyatt R, Robinson J, Sweet RW, Sodroski J, Hendrickson WA. Structure of an HIV gp120 envelope glycoprotein in complex with the CD4 receptor and a neutralizing human antibody. *Nature* **393**, 648-659 (1998).

6. Ozorowski G*, et al.* Open and closed structures reveal allostery and pliability in the HIV-1 envelope spike. *Nature* **547**, 360-363 (2017).

7. Gorman J*, et al.* Structures of HIV-1 Env V1V2 with broadly neutralizing antibodies reveal commonalities that enable vaccine design. *Nat Struct Mol Biol* **23**, 81-90 (2016).

8. Cale EM*, et al.* Virus-like Particles Identify an HIV V1V2 Apex-Binding Neutralizing Antibody that Lacks a Protruding Loop. *Immunity* **46**, 777-791 e710 (2017).

9. Liu Q*, et al.* Quaternary contact in the initial interaction of CD4 with the HIV-1 envelope trimer. *Nat Struct Mol Biol* **24**, 370-378 (2017).

10. Wang H*, et al.* Asymmetric recognition of HIV-1 Envelope trimer by V1V2 loop-targeting antibodies. *Elife* **6**, (2017).

11. Gorman J*, et al.* Structure of Super-Potent Antibody CAP256-VRC26.25 in Complex with HIV-1 Envelope Reveals a Combined Mode of Trimer-Apex Recognition. *Cell Rep* **31**, 107488 (2020).

12. Kong L*, et al.* Supersite of immune vulnerability on the glycosylated face of HIV-1 envelope glycoprotein gp120. *Nat Struct Mol Biol* **20**, 796-803 (2013).

13. Garces F*, et al.* Affinity Maturation of a Potent Family of HIV Antibodies Is Primarily Focused on Accommodating or Avoiding Glycans. *Immunity* **43**, 1053-1063 (2015).

14. Kong L*, et al.* Complete epitopes for vaccine design derived from a crystal structure of the broadly neutralizing antibodies PGT128 and 8ANC195 in complex with an HIV-1 Env trimer. *Acta Crystallogr D Biol Crystallogr* **71**, 2099-2108 (2015).

15. Barnes CO*, et al.* Structural characterization of a highly-potent V3-glycan broadly neutralizing antibody bound to natively-glycosylated HIV-1 envelope. *Nat Commun* **9**, 1251 (2018).

16. Fera D*, et al.* HIV envelope V3 region mimic embodies key features of a broadly neutralizing antibody lineage epitope. *Nat Commun* **9**, 1111 (2018).

17. Simonich CA*, et al.* Kappa chain maturation helps drive rapid development of an infant HIV-1 broadly neutralizing antibody lineage. *Nat Commun* **10**, 2190 (2019).

18. Kumar S*, et al.* A VH1-69 antibody lineage from an infected Chinese donor potently neutralizes HIV-1 by targeting the V3 glycan supersite. *Sci Adv* **6**, (2020).

19. Klein F*, et al.* Somatic mutations of the immunoglobulin framework are generally required for broad and potent HIV-1 neutralization. *Cell* **153**, 126-138 (2013).

20. Liao HX*, et al.* Co-evolution of a broadly neutralizing HIV-1 antibody and founder virus. *Nature* **496**, 469-476 (2013).

21. Zhou T*, et al.* Multidonor analysis reveals structural elements, genetic determinants, and maturation pathway for HIV-1 neutralization by VRC01-class antibodies. *Immunity* **39**, 245-258 (2013).

22. Zhou T*, et al.* Structural Repertoire of HIV-1-Neutralizing Antibodies Targeting the CD4 Supersite in 14 Donors. *Cell* **161**, 1280-1292 (2015).

23. Bonsignori M*, et al.* Maturation Pathway from Germline to Broad HIV-1 Neutralizer of a CD4-Mimic Antibody. *Cell* **165**, 449-463 (2016).

24. Jardine JG*, et al.* Minimally Mutated HIV-1 Broadly Neutralizing Antibodies to Guide Reductionist Vaccine Design. *PLoS Pathog* **12**, e1005815 (2016).

25. Scharf L*, et al.* Structural basis for germline antibody recognition of HIV-1 immunogens. *Elife* **5**, (2016).

26. Sajadi MM*, et al.* Identification of Near-Pan-neutralizing Antibodies against HIV-1 by Deconvolution of Plasma Humoral Responses. *Cell* **173**, 1783-1795 e1714 (2018).

27. Schommers P*, et al.* Restriction of HIV-1 Escape by a Highly Broad and Potent Neutralizing Antibody. *Cell* **180**, 471-489 e422 (2020).

28. Tolbert WD, Nguyen DN, Tehrani ZR, Sajadi MM, Pazgier M. Near-Pan-neutralizing, Plasma Deconvoluted Antibody N49P6 Mimics Host Receptor CD4 in Its Quaternary Interactions with the HIV-1 Envelope Trimer. *mBio* **12**, e0127421 (2021).

29. Dam KA*, et al.* HIV-1 CD4-binding site germline antibody-Env structures inform vaccine design. *Nat Commun* **13**, 6123 (2022).

30. Schorcht A*, et al.* The Glycan Hole Area of HIV-1 Envelope Trimers Contributes Prominently to the Induction of Autologous Neutralization. *J Virol* **96**, e0155221 (2022).
