# Supplementary material for: Structures and immune recognition of Env trimers from two Asia prevalent HIV-1 CRFs
Source: Nat Commun. 2023 Aug 4;14:4676. doi: 10.1038/s41467-023-40321-x (PMC10403546; doi:10.1038/s41467-023-40321-x)
Supplement: Supplementary file 3 — Reporting Summary [file 41467_2023_40321_MOESM3_ESM.pdf]

nature portfolio

Corresponding author(s): Bei Yang, Yiming Shao

Last updated by author(s): Jul 15, 2023

Reporting Summary

Nature Portfolio wishes to improve the reproducibility of the work that we publish. This form provides structure for consistency and transparency in reporting. For further information on Nature Portfolio policies, see our Editorial Policies and the Editorial Policy Checklist.

Statistics

For all statistical analyses, confirm that the following items are present in the figure legend, table legend, main text, or Methods section.

n/a	Confirmed	
☐	☒	The exact sample size (*n*) for each experimental group/condition, given as a discrete number and unit of measurement
☐	☒	A statement on whether measurements were taken from distinct samples or whether the same sample was measured repeatedly
☐	☒	The statistical test(s) used AND whether they are one- or two-sided<br>*Only common tests should be described solely by name; describe more complex techniques in the Methods section.*
☒	☐	A description of all covariates tested
☒	☐	A description of any assumptions or corrections, such as tests of normality and adjustment for multiple comparisons
☐	☒	A full description of the statistical parameters including central tendency (e.g. means) or other basic estimates (e.g. regression coefficient) AND variation (e.g. standard deviation) or associated estimates of uncertainty (e.g. confidence intervals)
☐	☒	For null hypothesis testing, the test statistic (e.g. *F*, *t*, *r*) with confidence intervals, effect sizes, degrees of freedom and *P* value noted<br>*Give P values as exact values whenever suitable.*
☒	☐	For Bayesian analysis, information on the choice of priors and Markov chain Monte Carlo settings
☒	☐	For hierarchical and complex designs, identification of the appropriate level for tests and full reporting of outcomes
☒	☐	Estimates of effect sizes (e.g. Cohen's *d*, Pearson's *r*), indicating how they were calculated

Our web collection on statistics for biologists contains articles on many of the points above.

Software and code

Policy information about availability of computer code

Data collection	Thermo Fisher Scientific TEM user interface and serialEM 3.7.3 for EM images collection;<br>Blu-Ice 5 for Crystallographic data collection;<br>Xcalibur 4.1 for Mass spectrometry data collection;<br>SparkControl MethodEditor V1.8;<br>EnSpireManager Version 3.
Data analysis	GraphPad Prism 8 and 9, MotionCor2, CTFFIND4.1.8, relion3.1.2, cryoSPARC V2.5 and V3.2, HKL2000, CCP4 7.0 and 8.0, Xia2-dials, PHENIX-1.19.2, PyMol 2.3.2, PDBePISA v1.52, R(3.6.1), Python(3.9.7), DeepEMhancer 0.14, crYOLO 1.6.1, Biacore Insight Evaluation Software 4.0.8.19879, Molprobity 4.02, Github repository: https://github.com/newjhon2013/HIV-project

For manuscripts utilizing custom algorithms or software that are central to the research but not yet described in published literature, software must be made available to editors and reviewers. We strongly encourage code deposition in a community repository (e.g. GitHub). See the Nature Portfolio guidelines for submitting code & software for further information.

Data

Policy information about availability of data

All manuscripts must include a data availability statement. This statement should provide the following information, where applicable:

- Accession codes, unique identifiers, or web links for publicly available datasets
- A description of any restrictions on data availability
- For clinical datasets or third party data, please ensure that the statement adheres to our policy

The cryoEM maps generated have been deposited in the Electron Microscopy Data Bank with accession numbers: EMD-34190 [https://www.ebi.ac.uk/emdb/EMD-34190], EMD-34192 [https://www.ebi.ac.uk/emdb/EMD-34192], EMD-34193 [https://www.ebi.ac.uk/emdb/EMD-34193], and EMD-34194 [https://www.ebi.ac.uk/emdb/EMD-34194]. The coordinates generated have been deposited to the Protein Data Bank under accession numbers 8GPK [https://doi.org/10.2210/pdb8GPK/pdb], 8GPI [https://doi.org/10.2210/pdb8GPI/pdb], 8GPJ [https://doi.org/10.2210/pdb8GPJ/pdb], 8GPG [https://doi.org/10.2210/pdb8GPG/pdb] and 8GP5 [https://doi.org/10.2210/pdb8GP5/pdb] respectively.
All other data needed to evaluate the conclusions in the paper are present in the paper, the Supplementary Materials and the Source Data file. Additional data related to this paper are available from the corresponding author upon reasonable request.
Previously published structures used in this study are available in the PDB with accession numbers: 5BMF [https://doi.org/10.2210/pdb5BMF/pdb], 5CJX [https://doi.org/10.2210/pdb5CJX/pdb], 5FYK [https://doi.org/10.2210/pdb5FYK/pdb], 5UM8 [https://doi.org/10.2210/pdb5UM8/pdb], 5FYJ [https://doi.org/10.2210/pdb5FYJ/pdb], 5FUU [https://doi.org/10.2210/pdb5FUU/pdb], 4TVP [https://doi.org/10.2210/pdb4TVP/pdb], 6P65 [https://doi.org/10.2210/pdb6P65/pdb], 5CCK [https://doi.org/10.2210/pdb5CCK/pdb]. Previously published CRF01_AE and CRF07_BC genome sequences mentioned in this study are available in the Genbank with accession numbers: AIS42911.1[https://www.ncbi.nlm.nih.gov/protein/AIS42911.1] and AIS42666.1[https://www.ncbi.nlm.nih.gov/protein/AIS42666.1].

Human research participants

Policy information about studies involving human research participants and Sex and Gender in Research.

Reporting on sex and gender	This information has not been collected.
Population characteristics	This information has not been collected.
Recruitment	This information has not been collected.
Ethics oversight	This information has not been collected.

Note that full information on the approval of the study protocol must also be provided in the manuscript.

Field-specific reporting

Please select the one below that is the best fit for your research. If you are not sure, read the appropriate sections before making your selection.

☒ Life sciences ☐ Behavioural & social sciences ☐ Ecological, evolutionary & environmental sciences

For a reference copy of the document with all sections, see nature.com/documents/nr-reporting-summary-flat.pdf

Life sciences study design

All studies must disclose on these points even when the disclosure is negative.

Sample size	No sample size calculation is performed. For cross-clades statistical analysis of V1 features, 323 clade A, 2322 clade B, 1577 clade C, 618 CRF01_AE and 56 CRF07_BC Env sequences that represents the fullest spectrum of corresponding subtypes in HIV database (https://www.hiv.lanl.gov) were used. For correlation analysis between V1 features and bNAbs neutralization sensitivities, all available neutralization datasets for related bNAbs and corresponding sequences in HIV database (https://www.hiv.lanl.gov) were used. As similar practices have also been implemented in previous publications, we believe the sample sizes are sufficient.
Data exclusions	No data was systematically excluded. The procedures of nsEM and cryo-EM data analysis involves software (relion3.1& cryoSPARC V2/V3) implemented sorting of particles that are damaged or are false-picked and are unlikely to refine correctly. For cross-clades statistical analysis of V1 features, Env sequences containing irregular characters (X, # and *) in V1 regions were excluded for accurate analysis. For correlation analysis between V1 features and bNAbs neutralization sensitivities, data without accurate IC50 values was excluded.
Replication	Experimental findings were reliably reproduced. All experiments were successfully repeated at least twice (mostly three times) on separate occasions.
Randomization	Randomization was not relevant to our study. Because there's no allocation of samples/organisms/participants involved in our study.
Blinding	Blinding was not relevant to our study. Because there's no allocation of samples/organisms/participants involved in our study.

Reporting for specific materials, systems and methods

We require information from authors about some types of materials, experimental systems and methods used in many studies. Here, indicate whether each material, system or method listed is relevant to your study. If you are not sure if a list item applies to your research, read the appropriate section before selecting a response.

Materials & experimental systems

n/a	Involved in the study
☐	☒ Antibodies
☐	☒ Eukaryotic cell lines
☒	☐ Palaeontology and archaeology
☒	☐ Animals and other organisms
☒	☐ Clinical data
☒	☐ Dual use research of concern

Methods

n/a	Involved in the study
☒	☐ ChIP-seq
☒	☐ Flow cytometry
☒	☐ MRI-based neuroimaging

Antibodies

Antibodies used	Recombinant antibodies ACS202, PG9, 8ANC195, VRC01, PGT121, 35O22, PGT145 and F6 were expressed and purified as outlined in the Methods section.<br>Antibodies from Human Immunodeficiency Virus type 1 (HIV-1) gp120 / Glycoprotein 120 ELISA Kit (Sino Biological, KIT11233, used at 1:1000)<br>Human Immunodeficiency Virus type 1 (HIV-1) gp120 / Glycoprotein 120 Antibody, Rabbit PAb (Sino Biological, 11233-RP02, used at 1:1000)<br>Anti-gp120/gp160 (pan) (Immune Tech, IT-001-002M18, used at 1:1000)
Validation	Amounts of antibodies were measured by NanoDrop. The purity and homogeneity of in-house antibodies were analyzed by size-exclusion chromatography and coomassie blue stained SDS-PAGE gels.<br>The quality control results of antibodies from Sino Biological are shown on the product websites (https://cn.sinobiological.com/elisa-kits/hiv-gp120-kit11233 & https://cn.sinobiological.com/antibodies/hiv-gp120-11233-rp02).<br>The quality control result of antibody from Immune Tech is shown on the product website (http://www.immune-tech.com.cn/product_show.php?id=114&tid=29)

Eukaryotic cell lines

Policy information about cell lines and Sex and Gender in Research

Cell line source(s)	FreeStyle™ 293-F suspension cells (Gibco, Cat.no. R79007)<br>ExpiCHO-S™ cells (Gibco, Cat.no. A29127)<br>Sf9 cells (Gibco, Cat.no. 11496015)<br>High Five Cells (Gibco, Cat.no.B85502)<br>TZM-bl cells (also called JC53BL-13) were obtained from the NIH AIDS Research and Reference Reagent Program (Cat. no. 8129)
Authentication	Not authenticated after purchase.
Mycoplasma contamination	Cell lines have been tested negative for mycoplasma contamination by PCR methods.
Commonly misidentified lines<br>(See ICLAC register)	No commonly misidentified cell lines were used.
